# Preparing Pharmacy Students for Practice to Support Underserved Older Persons

**DOI:** 10.1093/geroni/igaa057.2749

**Published:** 2020-12-16

**Authors:** Daniel Mansour

**Affiliations:** University of Maryland School of Pharmacy, Baltimore, Maryland, United States

## Abstract

Interprofessional collaboration is needed to ensure high quality care. Effective programs teaching necessary team-based care skills are under investigation. This study evaluated the Aging in Place program, an interprofessional practice experience (IPE), in preparing pharmacy students for practice with underserved older persons. The Assessment of Interprofessional Team Collaboration Scale (AITCS) and Team Decision Making Questionnaire (TCMQ) were administered to students before and after their experience. The number of disciplines represented, campuses involved, resident sessions, resident interactions, and health screenings performed were documented. Overall, AITCS and TDMQ scores improved after participation in the program. Since the program’s inception, there have been 7 disciplines represented, 2 campuses involved, 125 student participants, 2 housing buildings, 90 resident sessions, and 370 health screenings performed. The Aging in Place program has grown and shown that an IPE program is feasible to better prepare pharmacy students for collaborative care with older residents of affordable housing buildings.

